# Management of Bluetongue Outbreaks: The Practitioner’s Perspective

**DOI:** 10.3390/v18101163

**Published:** 2026-10-20

**Authors:** Olivier Salat

**Affiliations:** Veterinary Clinic Haute Auvergne, 15100 Saint Flour, France; olivier.salat@free.fr

**Keywords:** bluetongue, epidemiology, epidemic, management, vaccination

## Abstract

Since the early 2000s, Bluetongue has emerged within the European Union, and a few years later, the adaptation of Palearctic *Culicoides* species to the transmission of BTV infections marked a major shift in the epidemiology of this disease. Since then, epidemic waves have followed one another, with new serotypes continually appearing or the emergence of new variants being observed. The characteristics of these viruses vary depending on the serotype, but certain features are now well established. First, the duration of viremia—closely linked to the lifespan of erythrocytes in the different affected species—is prolonged and plays a major role in the reactivation of outbreaks from one year to the next. Its tropism for the reproductive system and its ability to cross the placental barrier have significant consequences for fertility and offspring survival. Knowledge of *Culicoides* biology has also improved considerably. Moisture and the presence of organic matter are sufficient for their development. Their abundance continues to increase, and they are only minimally susceptible to the insecticides commonly used in ruminants. Control of this vector therefore appears highly challenging. One positive aspect is that they rarely enter livestock buildings, and housing animals indoors—particularly in well-ventilated facilities—can be an effective supportive measure in controlling bluetongue. Fortunately, shortly after the emergence of the first outbreaks, vaccines were developed within a very short timeframe. Vaccination therefore remains the only truly effective means of controlling these infections. The conditions for their use have improved significantly. What is still lacking is a product covering the range of circulating serotypes in order to achieve optimal protection of our herds. However, this requires sustained surveillance, particularly for the early detection of new serotypes.

## 1. Introduction

Bluetongue (BT) is an infectious but non-contagious disease. It is an arboviral disease, meaning it is transmitted by arthropods—in this case, a biting midge of the genus *Culicoides* Latreille (*Diptera*: *Ceratopogonidae*). It affects ruminants, and certain forms can be particularly severe. The disease is caused by the bluetongue virus (BTV), a double-stranded RNA virus with a segmented genome (10 segments), belonging to the family *Sedoreoviridae* and the genus *Orbivirus*.

*Culicoides* spp. are small blood-feeding midges, reaching a maximum size of approximately 3 mm, and are therefore much smaller than mosquitoes. Their small size gives them a high capacity for wind-borne dispersal, allowing them to travel several tens of kilometers per day [1]. Approximately 1400 species have been recorded worldwide. Their larval development requires only organic matter and moisture, which explains their worldwide distribution, with the exception of Antarctica and New Zealand.

The female is hematophagous and takes a blood meal from humans or animals, with peak activity occurring at dusk. These insects can cause skin lesions when present in large numbers, but it is their role as vectors of arboviruses (Oropouche virus in humans, and orbiviruses and orthobunyaviruses in ruminants) that has made them particularly well known.

Historically [2,3], bluetongue was a tropical or subtropical disease; however, multiple BTV serotypes (BTV-1, BTV-2, BTV-4, BTV-9, and BTV-16) began circulating in southern Europe from the late 1990s onwards. Northwestern Europe, however, remained free of BTV until 2000 [4]. In October 2000, France was confronted with bluetongue for the first time [5]. The disease initially appeared in Corsica, where a BTV serotype 2 strain was identified one month after the presence of *Culicoides imicola* Kieffer had been confirmed.

In 2003, serotype BTV-4 emerged, followed by the identification of BTV-16 the following year, again in Corsica. In 2006, seven herds located along the northern border of mainland France were affected by infection with a new serotype, BTV-8, and over the following two years, the epidemic spread throughout the rest of the country. In 2008, an outbreak caused by BTV-1 originating from Spain began expanding from the Basque Country toward northeastern France.

Following large-scale compulsory vaccination campaigns, the country regained disease-free status in 2012. In Corsica, however, serotype BTV-1 emerged in 2013, followed in 2014 by BTV-27 (which is directly transmissible between goats). In 2015, a resurgence of BTV-8 occurred in the Allier department, with the infection spreading rapidly into neighboring areas of the Massif Central, although the clinical impact remained limited.

A bovine animal originating from Corsica was responsible for introducing BTV-4 serotype into the Alpine region, but its spread remained also limited. France was subsequently declared an endemic area for BTV-4 and BTV-8 infections. The latter spread again during 2019, albeit relatively silently.

A much more virulent serotype 8 variant emerged in Aveyron during the summer of 2023 and spread across the entire national territory within three years. At the same time, a new serotype, BTV-3, emerged in the Netherlands and spread to neighboring countries (including the United Kingdom). The years 2024 and 2025 saw its expansion across almost the entire French territory.

In 2024, a BTV-3 reassortant, distinct from the Dutch strain, circulated in Corsica and proved particularly pathogenic for sheep. It resulted from reassortment between two Sardinian strains: a non-virulent BTV-3 strain originating from North Africa and an autochthonous BTV-4 strain [6].

Thus, over the past 20 years, no fewer than four serotypes have circulated in mainland France (excluding Corsica), including three epidemic waves responsible for significant losses. Currently, infections with BTV-3, BTV-4, and BTV-8 serotypes are recognized as enzootic in France, and clinical cases must be reported. However, no restrictive measures (apart from those related to exportation) are associated with such reporting.

The characteristics of BTV infection are increasingly well understood, although they may vary between serotypes. The fact that Western countries have been affected has enabled a much better understanding of the disease, owing to the research resources deployed. Despite remaining uncertainties, many findings can already be applied in practice. What is now clear is that this disease can only be effectively controlled through vaccination.

## 2. A Virus That Continues to Surprise Us

### 2.1. An Unexpected Emergence

While a gradual northward spread of bluetongue cases had been expected, in parallel with the expansion of what was considered its sole vector in Mediterranean Europe, *C. imicola*, the year 2006 came as a real shock. It was then observed that Palearctic *Culicoides* species (*C. dewulfi* Goetghebuer, *C. obsoletus* Meigen) were capable of transmitting bluetongue viruses.

Two key realities emerged from this finding: first, that a disease long considered exotic could affect countries in Western Europe, and second, that a minute and widespread insect—previously largely overlooked—the *Culicoides* midge, was responsible for spreading the infection.

The second major event was that the disease, which had appeared during the summer of 2006 in the Maastricht region of the Netherlands, re-emerged the following summer after seemingly disappearing over the winter, and the epidemic subsequently spread rapidly. Thus, BTV-8 infection had overwintered.

Currently, six serotypes are present in Europe (1, 3, 4, 5, 8, and 12), with some reaching an endemic status in various countries. What remains surprising is that the conditions surrounding the initial introduction of BTV-8 and its adaptation to temperate European environments are still unknown.

However, these are not the only unresolved questions: Why do some infections remain sporadic and fail to spread? What is the origin of the BTV-8 variant that emerged in Aveyron in 2023, which is genetically very different from previously circulating strains? Why has the BTV-1 strain circulating in Spain not spread northward into France, as it did in 2008?

Much remains to be discovered regarding the conditions that lead to epidemics caused by this virus.

### 2.2. Diverse Clinical Manifestations

During the first BTV-8 epidemic, cattle were primarily affected, whereas the Aveyron variant involved both sheep and cattle, with more frequent peracute forms in the former. A similar pattern was observed during the BTV-3 epidemic. The same serotype, but associated with a completely different reassortant circulating simultaneously in Corsica, proved to be even more pathogenic for sheep.

In contrast, goats appeared less susceptible to infection with BTV serotypes transmitted by *Culicoides*. A marked variability in clinical impact between individuals has also been reported [7].

BTV causes severe damage to the endothelial cells of blood vessels, resulting in lesions of the mucous membranes (oral, nasal, and ocular), with discharges, congestion, edema, and hemorrhages associated with hyperthermia. More severe complications involving the upper respiratory tract and the esophagus may subsequently develop. Lameness associated with involvement of the coronary band is also observed [8].

Peracute forms with rapid fatal outcomes occur in sheep. In general, sheep are more severely affected than cattle, except during the 2006–2008 BTV-8 epidemic.

### 2.3. Prolonged Viremia

An important element in the epidemiology of these infections is the particularly prolonged duration of viremia. Indeed, it extends well beyond the appearance of antibodies in the bloodstream (Table 1) [1]. This phenomenon is explained by the localization of the virus within membrane invaginations of erythrocytes.

This property is essential, as it largely accounts for the re-emergence of the disease from one year to the next, despite periods of vector inactivity. However, it is important to distinguish the duration of infectious viremia, which can last up to two months in cattle, from the period during which molecular testing remains positive, which can extend well beyond six months [9,10,11] (Table 2). Thus, PCR tests performed on blood from infected animals may remain positive for 28 weeks, with high Ct values observed for up to 15 weeks [12].

### 2.4. Transplacental and Semen Transmission

It has also been demonstrated that this virus exhibits a particular tropism for the reproductive system, along with, for certain serotypes, the ability to cross the placental barrier. This results in several consequences:

- Impairment of male fertility, potentially affecting one to two spermatogenic cycles, resulting in infertility lasting 3 to 6 months [13,14,15,16];
- Impairment of female fertility: a fivefold increase in the relative risk of return to estrus in infected cows, along with an increase in the interval between the first and final artificial insemination (duration multiplied by 2.5 in infected cows) [17,18];
- Possible transmission via semen as well as through infected embryos. This property has so far only been demonstrated for serotype 8 [5,19]; The use of frozen semen collected on-farm in 2008 is thought to have contributed to the re-emergence of BTV-8 infection in the Allier region in 2015 [20];
- Abortions, mainly occurring between 3 and 7 months of gestation, but potentially up to full term [21];
- The birth of malformed calves: these malformations primarily affect the central nervous system (CNS) (hydranencephaly, hydrocephalus, focal necrotizing meningoencephalitis) in ovine fetuses between 50 and 100 days of gestation and in bovine fetuses between 80 days and mid-gestation. In bovine fetuses, focal CNS lesions have also been detected even beyond mid-gestation [22];
- The birth of viremic calves, mainly associated with infections occurring during the second half of gestation [17]. This viremia persists for several weeks but has never been detected beyond 5 months [23].

### 2.5. Continuing Emergence of New Threats

The routes of introduction of BTV infections into Europe can be classified into three main pathways: Spain and Sardinia, reflecting trans-Mediterranean transmission routes from North Africa, as well as the Netherlands. For the latter route, the mechanism of introduction remains unknown.

In addition to this almost constant risk, there is the potential for mutations of endemic serotypes, as observed during the BTV-8 epidemic wave in 2023. It is therefore particularly difficult to predict the origin, cause, and dynamics of future outbreaks. This highlights the fundamental importance of ongoing epidemiological surveillance.

## 3. Key Lessons from Epidemic Waves

### 3.1. The Potential Impact of Climate Change

One cannot help but draw parallels between the emergence of BTV in Europe and climate change: indeed, peaks in infection often coincide with heatwave episodes, and the fact that diverse strains emerge from multiple locations may support this hypothesis, particularly in the absence of major changes in cattle movements [5,24,25]. However, these epidemiological associations are not demonstrated causal relationships and a cautious approach is warranted before definitively establishing a link between these two events. Moreover, trade and animal density are two factors that have contributed to the introduction and spread of these arboviruses [26].

When examining the distribution of *C. imicola* in France since the early 2000s, its population remains confined to the coastal areas of the Var and Alpes-Maritimes regions. Only its density appears to be increasing [27].

It is rather the Palearctic species that seem to have been affected by climate change. *Culicoides* populations have been observed to increase during warm periods, and more importantly, their seasonal activity periods have lengthened, contributing to the persistence of epidemics from one year to the next.

### 3.2. Risk Factors

In the study conducted in the Netherlands [28], the following risk factors were identified:

- Grazing (whether for short or extended periods);
- Introduction of cattle from epidemic areas;
- Presence of sheep on the farm;
- The timing of the first reported case in the area.

Keeping animals outdoors increases their exposure to *Culicoides* bites, particularly at dawn and dusk, which correspond to peak vector activity periods [29].

Regarding the second risk factor, it was observed that the restriction of animal movements implemented in 2006 in the Netherlands likely slowed, but unfortunately did not prevent, the spread of infection [30].

Finally, the later a region is affected during the season, the closer it is to the period of vector inactivity, and consequently, the lower the overall impact (personal observation).

In the aforementioned study, factors associated with a reduced impact of bluetongue included housing animals in buildings with large ventilation openings and the use of mechanical ventilation: airflow within buildings appears to interfere with *Culicoides* feeding behavior.

The use of insecticides was not associated with serological prevalence. The limited effectiveness of insecticides has already been confirmed by the EFSA Panel on Animal Health and Welfare [31].

### 3.3. Bluetongue: An Uncontrollable Spread

Two elements explain this situation:

(i) It is a vector-borne disease transmitted by *Culicoides*, insects whose proliferation is particularly difficult to control: their salivary accessory glands constitute the primary site of BTV replication. Viral titers vary depending on the serotype studied (higher for BTV-3 compared with BTV-8) [32].

During the BTV-3 epidemic in the Netherlands, 40.5% of the tested pools of captured *Culicoides* were found to be virus carriers, with a broader range of vector species involved, including *C. scoticus* Downes & Kettle, *C. obsoletus*, *C. dewulfi*, *C. chiopterus* Meigen, and *C. punctatus* Meigen [33], which may explain the rapid spread of the epidemic due to the abundance of these species. Moreover, a single *Culicoides* can simultaneously transmit two BTV serotypes [34]. This vector cannot be controlled on a large scale and can be carried by the wind over long distances (up to 700 km—as illustrated by the introduction of BTV-3 into the United Kingdom, or into Northern Ireland via the Isle of Man) [5]. Above all, the *Culicoides* midge requires only organic matter and moisture to complete its life cycle.

The importation of infected *Culicoides* was suspected during the first emergence of bluetongue in Europe in 2006, particularly via cut flowers originating from South Africa. This hypothesis is now considered highly improbable [35]. It appears that international transport of humans or goods does not represent a risk for the introduction of exotic *Culicoides* species; however, no definitive conclusions have yet been reached regarding the transport of live animals.

Keeping animals indoors greatly reduces their exposure to infection. Strengthening vector control measures within animal housing facilities could further reduce contact between *Culicoides* midges and susceptible hosts. For example, it has been demonstrated that installing insecticide-impregnated screens on stable doors can reduce their entry [36]. In contrast, a study [37] showed that the effectiveness of permethrins applied to animals is very limited, not to mention their ecotoxicity. There is currently no evidence supporting their effectiveness in reducing BTV transmission.

(ii) Moreover, the global trade in live animals has increased steadily over the past 20 years, meaning that the risk of importing animals carrying BTV has never been greater [5]. Restrictions on animal movements, as implemented during the first bluetongue epidemic in 2006–2008, helped to slow the spread of the disease but did not prevent it altogether.

### 3.4. Herd Immunity: A Very Gradual Establishment

We were affected by the resurgence of a new BTV-8 variant during the summer of 2023 in the Aveyron department. As our client base is located in a neighboring department (Cantal), we rapidly observed clinical signs a few weeks after the alert, i.e., toward the end of August 2023 (personal observation). The increasing number of cases observed until late in the season (end of November) led us to believe that the wave had subsided.

However, as early as August 2024, the epidemic resumed with even greater intensity and reached a larger scale than the previous year, despite significantly higher vaccination coverage. This observation is consistent with that reported in the Netherlands in 2023, which was the subject of a publication [28].

In a survey conducted at the beginning of 2024, based on the detection of antibodies in bulk tank milk, while the epidemic had affected almost the entire country, the herd-level prevalence was 64%. Based on the positivity levels observed in bulk tank milk, the average within-herd prevalence was estimated at 36%. This allowed an overall prevalence of 19% to be estimated at the level of the cattle population in the Netherlands. Even in regions where nearly all herds had been affected by the infection (99% herd prevalence), only an average of 45% of cattle showed antibodies, which remains well below the commonly accepted herd immunity threshold of 70% [38].

### 3.5. How to Limit Its Impact

There is a clear benefit in reducing exposure to vectors by keeping herds indoors. This practice has proven effective not only against Schmallenberg virus and BTV-8 [30,39], but also against BTV-3 [28]. It is highly likely that animals kept permanently indoors are less exposed to *Culicoides* bites [40].

However, *Culicoides* midges have been found inside buildings [41], meaning that this protection cannot be complete. Nevertheless, the protective effect of housing animals indoors has been clearly demonstrated [42,43].

This has been observed, for example, in zero-grazing robotic milking systems, where only dry cows—those that were kept at pasture—were affected (personal observation).

## 4. Control Measures: Vaccination as the Only Effective Strategy

### 4.1. Choice of Vaccine Type

Live attenuated vaccines represent a persistent risk of recombination. For instance, in November 2008, limited circulation of a BTV-11 strain was detected in Belgium. The genome segment 2 of this virus was found to be identical to that of a South African attenuated vaccine strain. This provided clear evidence of a reversion to virulence, albeit limited, of this attenuated strain [44].

This risk, combined with the possibility of recombination between attenuated vaccine strains and wild-type viruses [45], has led to the exclusive authorization of inactivated vaccines in Europe.

Vaccines are available against various serotypes, some of which are bivalent (e.g., 1+8, 4+8). The protection conferred by vaccination against a given serotype is generally serotype-specific, and cross-protection against other serotypes remains very limited and incomplete [46,47,48].

### 4.2. Vaccine Efficacy

Epidemic waves of bluetongue have created enormous pressure on policymakers and pharmaceutical companies to implement emergency vaccination programs. The responsiveness of vaccine manufacturers proved critical in providing solutions to curb these outbreaks and their considerable economic impact. Vaccine development therefore became a race against time, and their commercialization was accelerated, most often with safety as the primary guarantee. As a result, questions were raised regarding their actual efficacy, particularly for the most recently developed vaccines. However, with regard to BTV-8, the emergence in 2023 of a new variant that was genetically entirely different from the original strain did not compromise the preventive effectiveness of vaccination (personal observation).

Studies assessing vaccine efficacy can be conducted at several levels:

- First, the production of antibodies induced by vaccination can be quantified; these may be total antibodies, or, more specifically, the production of virus-neutralizing antibodies, which are better indicators of protective immunity. This represents the lowest level of evidence.
- Vaccine efficacy can also be assessed through experimental challenge studies. This requires the ability to reproduce the disease under experimental conditions. In such cases, a group of animals—typically small in number—is vaccinated according to a defined protocol, while a control group of equal size remains unvaccinated. Then, a few days after effective immunity has been established, both groups are subjected to an experimental challenge. The effects of the vaccine are then evaluated based on the difference in clinical impact of the experimental infection between the two groups. This represents the second level of evidence of vaccine efficacy, superior to the previous one.
- Finally, the third level of evidence—also the highest—is the field trial. This involves larger sample sizes and includes several farms naturally exposed to a high risk of the disease for which the vaccine is being tested. Within each farm, two comparable groups of animals are constituted. This randomization is essential for the validity of the trial. Indeed, the identified risk factors for the disease must be exactly the same in both groups; otherwise, no valid comparison can be made. For example, in the case of bluetongue, if one group is kept indoors while the other remains outdoors, their level of exposure to risk is clearly not comparable. The same applies when comparing two different farms, one vaccinated and the other not: the number of potential confounding variables (feeding, herd management practices, exposure to infectious risks, overall herd immunity) is so large that meaningful comparisons cannot be made. When very large sample sizes are included, some sources of variation may be mitigated; however, it remains essential to ensure that there are no major differences in farm typology or geographical conditions.

While there has been little controversy regarding the effectiveness of vaccination against the initial circulating BTV serotypes (8, 1, and 4), the results of vaccination campaigns against the recent BTV-3 epidemic have generated somewhat more debate.

We have access to studies conducted in the Netherlands that help provide some clarification. The non-systematic use of vaccination did not allow the epidemic to be halted; for example, only 57% of Dutch sheep farmers implemented vaccination, which was primarily performed using SYVAZUL^®^BTV3 due to its early availability [49].

When comparing health outcomes between vaccinated and unvaccinated farms, adult mortality was 1.5 times higher in unvaccinated flocks, while lamb mortality was 1.22 times higher. Objectively, the effectiveness of vaccination is therefore indisputable [49].

Another study attempted to compare the effects of the three commercial vaccines (BULTAVO^®^ 3, SYVAZUL^®^BTV3, BLUEVAC^®^3), available against BTV-3 infection [12]. However, this study presents too many biases—lack of randomization, statistically uneven distribution with regard to grazing practices, absence of data on the timing of vaccination, and highly questionable comparison criteria—for its results to be considered reliable.

Nevertheless, this does not imply that these vaccines have equivalent efficacy. Rather, it highlights the current lack of robust, objective data needed to draw meaningful conclusions on this matter.

What is certain, however, is that vaccination against BTV-3 contributed to reducing the health impact of the epidemic in the Netherlands in 2024 [5].

### 4.3. Vaccine Use: Major Advances

We have encountered recurring issues regarding vaccine availability. This quickly raised the question of whether switching between commercial products targeting the same serotype could affect vaccine efficacy.

It has been demonstrated that, for certain vaccines—such as SYVAZUL^®^ 4+8 and BTVPUR^®^ 4+8 [50], as well as vaccines targeting serotype 3 (unpublished BI data)—a booster vaccination directed against the same serotype(s) using a heterologous vaccine remains effective.

Furthermore, it has been shown that administering multiple vaccines on the same day (against BTV3 and EHDV) does not impair the development of robust immunity against each valence [51].

These findings provide welcome flexibility in the practical implementation of vaccination protocols.

### 4.4. Future Perspectives

It appears that the only long-term solution for controlling these BTV epidemic waves lies in the widespread use of inactivated multivalent vaccines covering the various serotypes circulating in Europe.

In this context, the recent development of a vaccine referred to as DISA (Disabled Infectious Single Animal) represents a promising new platform, based on the deletion of genome segment 10 of the bluetongue virus (BTV), achieved through reverse genetics [52,53,54].

From this platform, multiple serotypes can be combined into a single vaccine dose by exchanging the viral proteins VP2 and VP5 from different serotypes onto a common backbone. Initial trials have shown that it can induce strong immunity and that the virus cannot be transmitted by *Culicoides*.

Furthermore, it allows differentiation between vaccinated animals and those infected with wild-type virus (DIVA strategy) [55,56].

## 5. Conclusions

In January 2026, the European Union decided to reclassify Bluetongue, moving it to categories D + E, whereas it had previously been classified as C + D + E, with this change taking effect from July 2026. This decision is driven by the abundance of vectors as well as the multiplicity of serotypes (currently six) circulating within the European Union. While fully understandable, this decision may have adverse consequences on the surveillance of vectors and viral serotypes, potentially leading to a decline in responsiveness.

The epidemic waves of BTV infections, from which we were previously free, now threaten the survival of our farming systems. Over the past 20 years, their economic cost (both direct and indirect losses) has continued to increase. It is now clear that these infections can currently only be controlled through vaccination. Significant progress has been made in this field, and access to vaccines covering more than two serotypes should soon become a reality. In this regard, one euro invested in vaccination can yield savings of up to 13 to 14 euros [57]. Responsibility for vaccination now lies with the farmer. As veterinarians, we can only make every effort to strongly encourage them to vaccinate.

## Figures and Tables

**Table 1:** Mean duration of BTV Viremia in cattle and sheep.

Species	Mean Duration of Viremia	95% Credible Interval
Cattle	20.53 days	18.76–22.26 days
Sheep	16.10 days	14.12–18.20 days

**Table 2:** Duration of BTV infectious Viremia and positive RTqPCR according to affected species.

	Duration of Infectious Viremia	Duration of Positive RTqPCR
Cattle	7–63 days	Up to 250 days
Sheep	30 days	4–5 months
Goats	30 days	2 months

## Data Availability

No new data were created or analyzed in this study. Data sharing is not applicable to this article.
